# Loss of *TSC1/TSC2* sensitizes immune checkpoint blockade in non–small cell lung cancer

**DOI:** 10.1126/sciadv.abi9533

**Published:** 2022-02-04

**Authors:** Qingyuan Huang, Fei Li, Hai Hu, Zhaoyuan Fang, Zhendong Gao, Guozhan Xia, Wai-Lung Ng, Alireza Khodadadi-Jamayran, Ting Chen, Jiehui Deng, Hua Zhang, Christina Almonte, Kristen Labbe, Han Han, Ke Geng, Sittinon Tang, Gordon J. Freeman, Yuan Li, Haiquan Chen, Kwok-Kin Wong

**Affiliations:** 1 Department of Thoracic Surgery and State Key Laboratory of Genetic Engineering, Fudan University Shanghai Cancer Center, Shanghai, China.; 2 Institute of Thoracic Oncology, Fudan University, Shanghai, China.; 3 Department of Pathology, School of Basic Medical Sciences, Fudan University, Shanghai, China.; 4 Laura and Isaac Perlmutter Cancer Center, New York University Grossman School of Medicine, NYU Langone Health, New York, NY, USA.; 5 State Key Laboratory of Cell Biology, Innovation Center for Cell Signaling Network, CAS Center for Excellence in Molecular Cell Science, Shanghai Institute of Biochemistry and Cell Biology, Chinese Academy of Sciences, University of Chinese Academy of Sciences, Shanghai, China.; 6 School of Pharmacy, Faculty of Medicine, The Chinese University of Hong Kong, Sha Tin, Hong Kong SAR, China.; 7 Applied Bioinformatics Laboratories and Genome Technology Center, Division of Advanced Research Technologies, New York University Langone Medical Center, New York, NY, USA.; 8 Department of Medical Oncology, Dana-Farber Cancer Institute, Boston, MA, USA.; 9 Department of Medicine, Brigham and Women’s Hospital, Harvard Medical School, Boston, MA, USA.; 10 Department of Pathology, Fudan University Shanghai Cancer Center, Shanghai, China.

## Abstract

Tuberous sclerosis complex subunit 1 (*TSC1*) and 2 (*TSC2*) are frequently mutated in non–small cell lung cancer (NSCLC), however, their effects on antitumor immunity remained unexplored. A CRISPR screening in murine *Kras^G12D^*/*Trp53^−/−^* (KP) model identified *Tsc1* and *Tsc2* as potent regulators of programmed cell death ligand 1 (Pd-l1) expression in vitro and sensitivity to anti–programmed cell death receptor 1 (PD-1) treatment in vivo. *TSC1* or *TSC2* knockout (KO) promoted the transcriptional and membrane expression of PD-L1 in cell lines. *TSC2*-deficient tumors manifested an inflamed microenvironment in patient samples and The Cancer Genome Atlas dataset. In syngeneic murine models, KP-*Tsc2*-KO tumors showed notable response to anti–PD-1 antibody treatment, but *Tsc2*–wild-type tumors did not. Patients with *TSC1*/*TSC2*-mutant NSCLC receiving immune checkpoint blockade (ICB) had increased durable clinical benefit and survival. Collectively, *TSC1*/*TSC2* loss defines a distinct subtype of NSCLC characterized as inflamed tumor microenvironment and superior sensitivity to ICB.

## Teaser

*TSC1*/*TSC2* loss defines a distinct subtype of NSCLC with inflamed tumor microenvironment and superior sensitivity to ICB therapy.

## INTRODUCTION

Therapeutic blockade of the programmed cell death receptor 1 (PD-1) and programmed cell death ligand 1 (PD-L1) pathway has been demonstrated to bring durable antitumor response by clinical trials and transformed the treatment of a wide range of advanced cancers, including non–small cell lung cancer (NSCLC) (*1*). However, only a minority (less than 20%) of unselected patients with NSCLC could benefit from anti–PD-1/PD-L1 immunotherapy, while 20 to 40% of these patients suffered severe adverse events (*2*). It is of vital significance to identify patients who are more likely to benefit from immune checkpoint blockade (ICB) and to maximize efficacy and minimize toxicity.

Currently, PD-L1 expression is the only Food and Drug Administration–approved predictive biomarkers for NSCLC (*1*). Our previous study showed that although greatest survival benefit was observed in patients with PD-L1–strong positive NSCLC, ICB therapy also significantly improved the survival of PD-L1–negative NSCLC (*2*). The findings indicated that PD-L1 expression was an imperfect biomarker. In addition, somatic mutations of several specific oncogenes and tumor suppressor genes (TSGs), including *EGFR*, *KRAS*, and *P53*, have been proven to be associated with treatment response to ICB therapy. Actually, genetic variants of tumor cells could modulate the host antitumor immune response via multiple mechanisms, such as modulating PD-L1 expression and reprogramming tumor microenvironment (TME) (*3*–*5*).

To identify previously unidentified immune modulators, we performed a CRISPR screening in a murine *Kras^G12D^*/*Trp53^−/−^* (KP) lung cancer model (C57BL/6 background) (*6*) and found that tuberous sclerosis complex subunit 1 (*Tsc1*) and subunit 2 (*Tsc2*) as potent regulators of Pd-l1 expression and sensitivity to PD-1 blockade treatment. *TSC1*/*TSC2* mediate the cross-talk between phosphatidylinositol 3-kinase–AKT and liver kinase B1–adenosine 5′-monophosphate–activated protein kinase pathways, suppress mammalian target of rapamycin complex 1 (mTORC1) signaling via the small guanosine triphosphatase Rheb, and function as tumor suppressors (*7*). Inactivating mutations of *TSC1*/*TSC2* cause tuberous sclerosis, an autosomal dominant disorder, and also frequently occur in many human cancers, including NSCLC (*8*). Here, we used lung cancer cell lines, patient samples, The Cancer Genome Atlas (TCGA) databases, and syngeneic murine models to comprehensively investigate the clinical relevance between *TSC1*/*TSC2* status and the immune characteristics of NSCLC and the efficacy of ICB therapy in *TSC1*/*TSC2*-deficent NSCLC.

## RESULTS

### In vitro and in vivo screening identified *Tsc1*/*Tsc2* as candidates of immune modulators in NSCLC

**Fig. 1.**
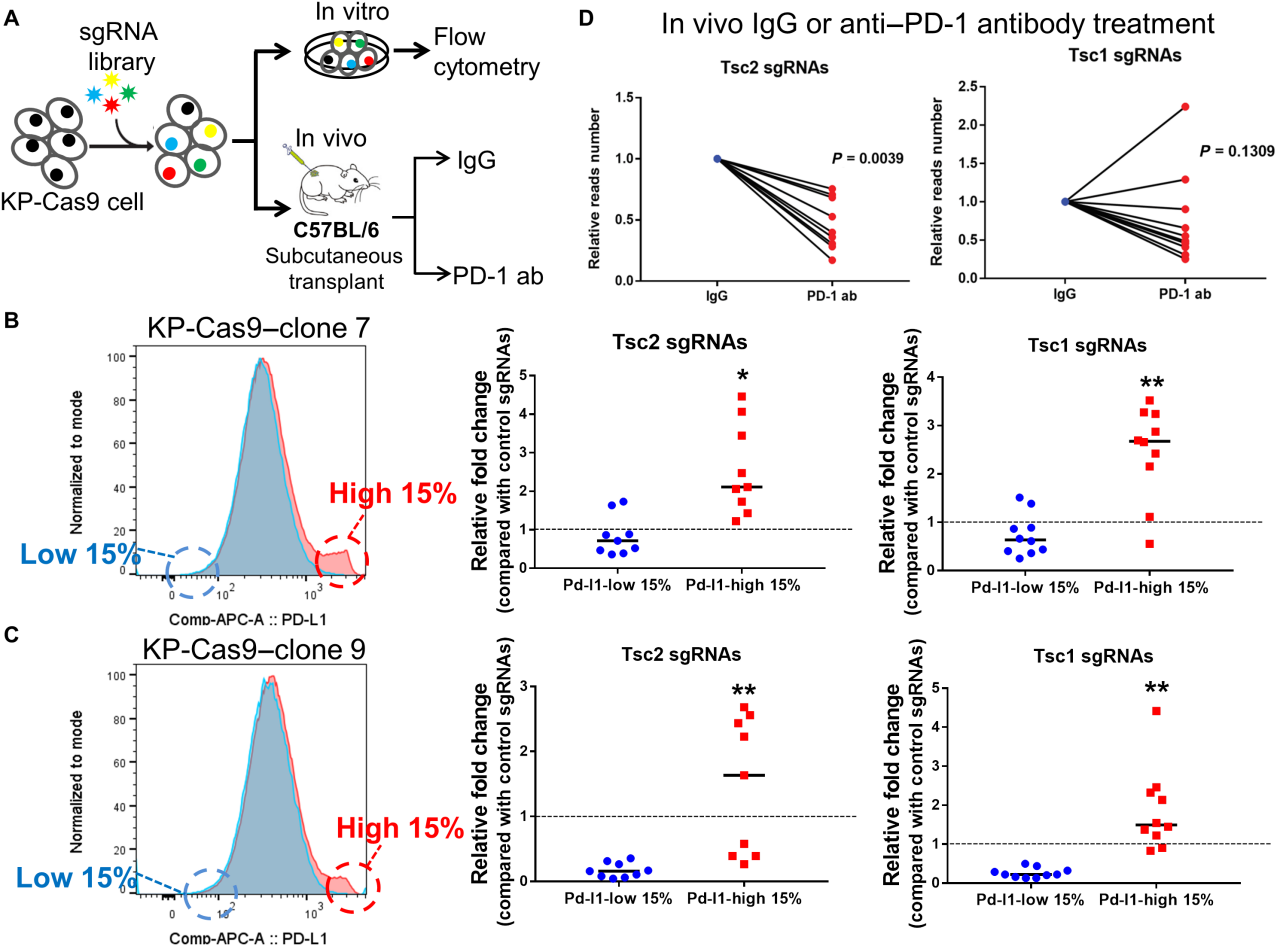
In vitro and in vivo CRISPR screening identified Tsc1/Tsc2 as candidates of immune modulators. (**A**) Diagram of CRISPR screening system. ab, antibody. (**B** and **C**) In vitro screening in KP-Cas9 clone 7 (B) and KP-Cas9 clone 9 (C). The top 15% Pd-l1–high and the top 15% Pd-l1–low populations were sorted, and sgRNAs of *Tsc2* and *Tsc1* were enriched in Pd-l1–high population. Blue peak, cells transfected with empty vector; red peak, cell pools transfected with sgRNA library. (**D**) Relative reads number of *Tsc2* and *Tsc1* in the in vivo screening in KP-Cas9 clone 7. Twelve tumors from six mice were included in each group of the screen. *P < 0.05 and **P < 0.01. APC, allophycocyanin.

To systemically evaluate cell-intrinsic regulators of antitumor immunity, we developed an in vitro and in vivo CRISPR screening using the murine KP lung cancer model (Fig. 1A). KP cells were engineered to express Cas9, and two clonal KP-Cas9 cell lines (clones 7 and 9) were selected to provide genetic and cellular homogeneity for subsequent screens, as described previously (*6*). After the KP-Cas9 clones were transfected with single-guide RNA (sgRNA) library and passaged in vitro, an obvious shift of PD-L1 expression was observed in flow cytometry, and the top 15% PD-L1–high populations and top 15% PD-L1–low populations were sorted (Fig. 1, B and C). Through next-generation sequencing (NGS), we found that *Tsc1* and *Tsc2* were among the top hits whose sgRNAs were significantly depleted in PD-L1–low populations and enriched in PD-L1–high populations (*P* < 0.05; Fig. 1, B and C), indicating that the loss of *Tsc1* or *Tsc2* potentially promoted PD-L1 expression.

For in vivo screening, immunocompetent C57BL/6 mice bearing KP-Cas9 clone 7 were treated with PD-1 antibody or isotype control (ctrl) since day 7 (three times per week). After 2 weeks, 12 tumors from six mice in each group were harvested for amplicon sequencing. Compared with isotype control, sgRNAs of *Tsc2* were significantly depleted in the PD-1 antibody group (*P* = 0.0039), and sgRNAs of *Tsc1* were modestly depleted in depleted (*P* = 0.13; Fig. 1D), indicating that *Tsc2*-loss tumors might be vulnerable to ICB therapy.

### *TSC2* deficiency up-regulates PD-L1 expression in lung cancer cell lines

**Fig. 2.**
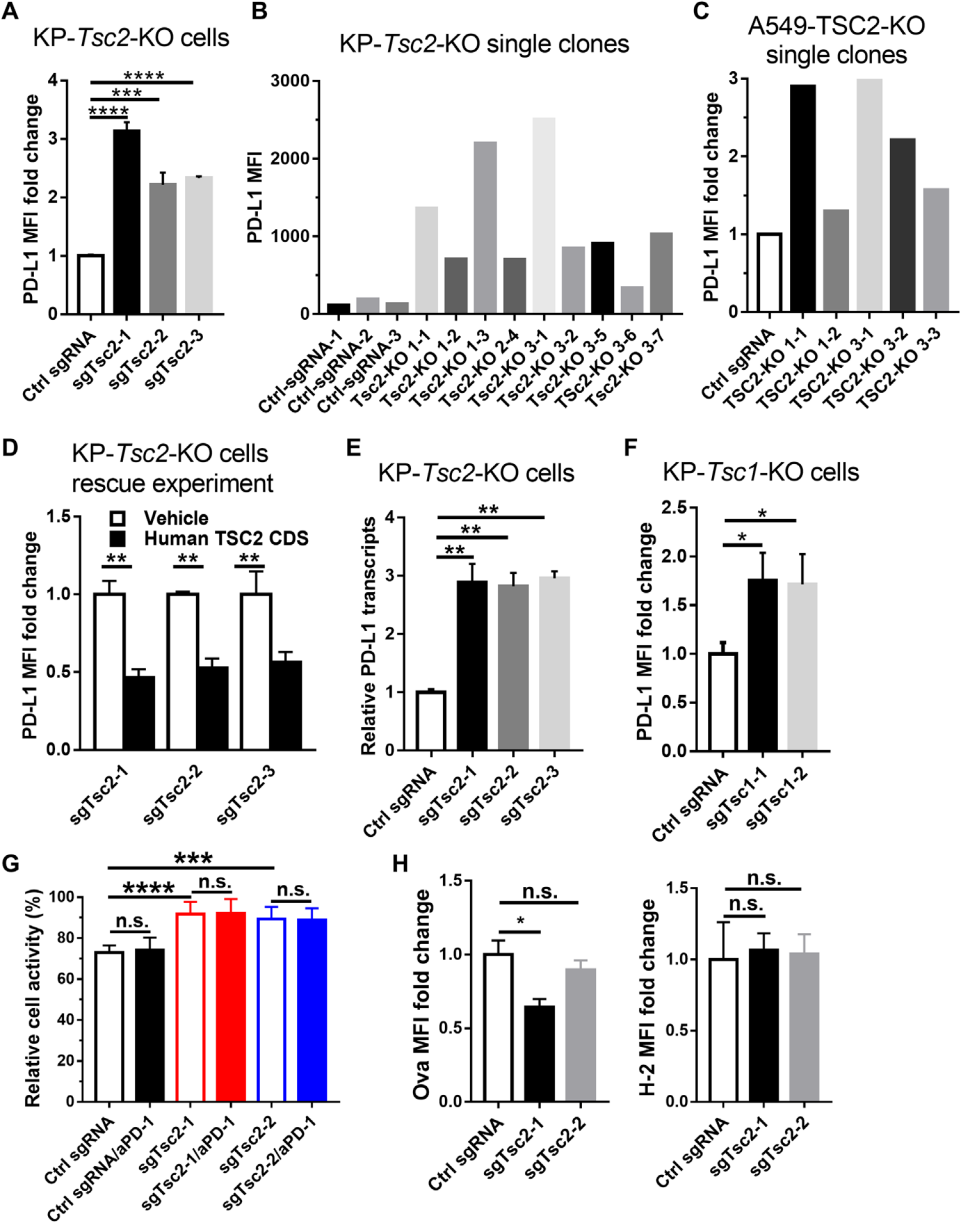
*Tsc2*-KO promoted PD-L1 expression in lung cancer cells. (**A**) Membrane PD-L1 expression in KP-Cas9 cells transfected with sgRNA for Ctrl or *Tsc2*. (**B**) Membrane PD-L1 expression in clones of KP-*Tsc2*-KO cells in (A). (**C**) Membrane PD-L1 expression in clones of human A549-*TSC2*-KO cells. (**D**) Membrane PD-L1 expression in KP-*Tsc2*-KO cells transfected with vehicle or human *TSC2* coding sequence (CDS). (**E**) Real-time PCR analyses of Pd-l1 mRNA level in KP-Cas9 cells transfected with sgRNA for Ctrl or *Tsc2*. (**F**) Membrane PD-L1 expression in KP-Cas9 cells transfected with sgRNA for Ctrl or *Tsc1*. (**G**) *Tsc2*-KO in KP-Ova cells significantly inhibited the cell-killing activity of OT-1 T cells in the in vitro killing assay, but the addition of anti–PD-1 antibody could not reverse this inhibition. (**H**) Flow cytometry showed that *Tsc2*-KO did not increase the membrane level of Ova and H-2 in the in vitro killing assay. Data are shown as means ± SEM. *P < 0.05, **P < 0.01, ***P < 0.001, and ****P < 0.0001. n.s., not significant; aPD-1, anti–PD-1 antibody.

To confirm the findings of in vitro CRISPR screening, we transfected lung cancer cell lines with individual sgRNAs of *Tsc1 or Tsc2*. Flow cytometry analyses showed that the depletion of *Tsc2* significantly increased membrane Pd-l1 expression level in KP cells (*P* < 0.001; Fig. 2A and fig. S1, A to C). We also established clonal *Tsc2*–knockout (KO) KP cell lines with the three individual sgRNAs and confirmed the efficiency of genome editing via Sanger sequencing (fig. S2, A to I). Flow cytometry analyses showed that the Pd-l1 expression in the *Tsc2*-KO KP clones was higher than those transfected with Ctrl sgRNAs (Fig. 2B). *TSC2*-loss mediated up-regulation of PD-L1 expression was also validated in human A549 lung cancer cell line (Fig. 2C and fig. S3, A to E). Next, we rescued the KP-*Tsc2*-KO cell lines by transfecting the human *TSC2* coding sequences and found that the expression level of Pd-l1 was significantly inhibited after reexpression of human TSC2 (Fig. 2D and fig. S4). Furthermore, quantitative realtime polymerase chain reaction (qRT-PCR) analyses revealed that the Pd-l1 mRNA levels of KP-*Tsc2*-KO cells were significantly higher than that of control cells (*P* < 0.01; Fig. 2E), indicating that *Tsc2* regulated Pd-l1 expression at transcriptional level. Depletion of the expression of *Tsc1* in KP cells also significantly increased the expression of Pd-l1 (Fig. 2F and fig. S5).

We further constitutively expressed neoantigen ovalbumin in KP (KP-Ova) cell line, depleted the expression of *Tsc2* (fig. S6), and then cocultured with OT-1 T cells. This in vitro killing assay showed that OT-1 T cells exerted a significantly reduced cytotoxicity to *Tsc2*-KO KP cells than *Tsc2*–wild-type (WT) cells; however, this inhibition of cytotoxicity could not be reversed by the addition of anti–PD-1 antibody in the coculture system (Fig. 2G). Besides, flow cytometry showed that depletion of *Tsc2* in KP cells did not increase the tumor antigen expression (Ova) or presentation via major histocompatibility complex molecules (H-2) in the coculture system (Fig. 2H). These findings indicate that the mechanism might not be mediated by the PD-1/PD-L1 axis or by neoantigen presentation, and future investigations are warranted.

### *TSC2* loss correlates with the expression of immune checkpoint molecules and confers poor prognosis in patients with NSCLC

**Fig. 3.**
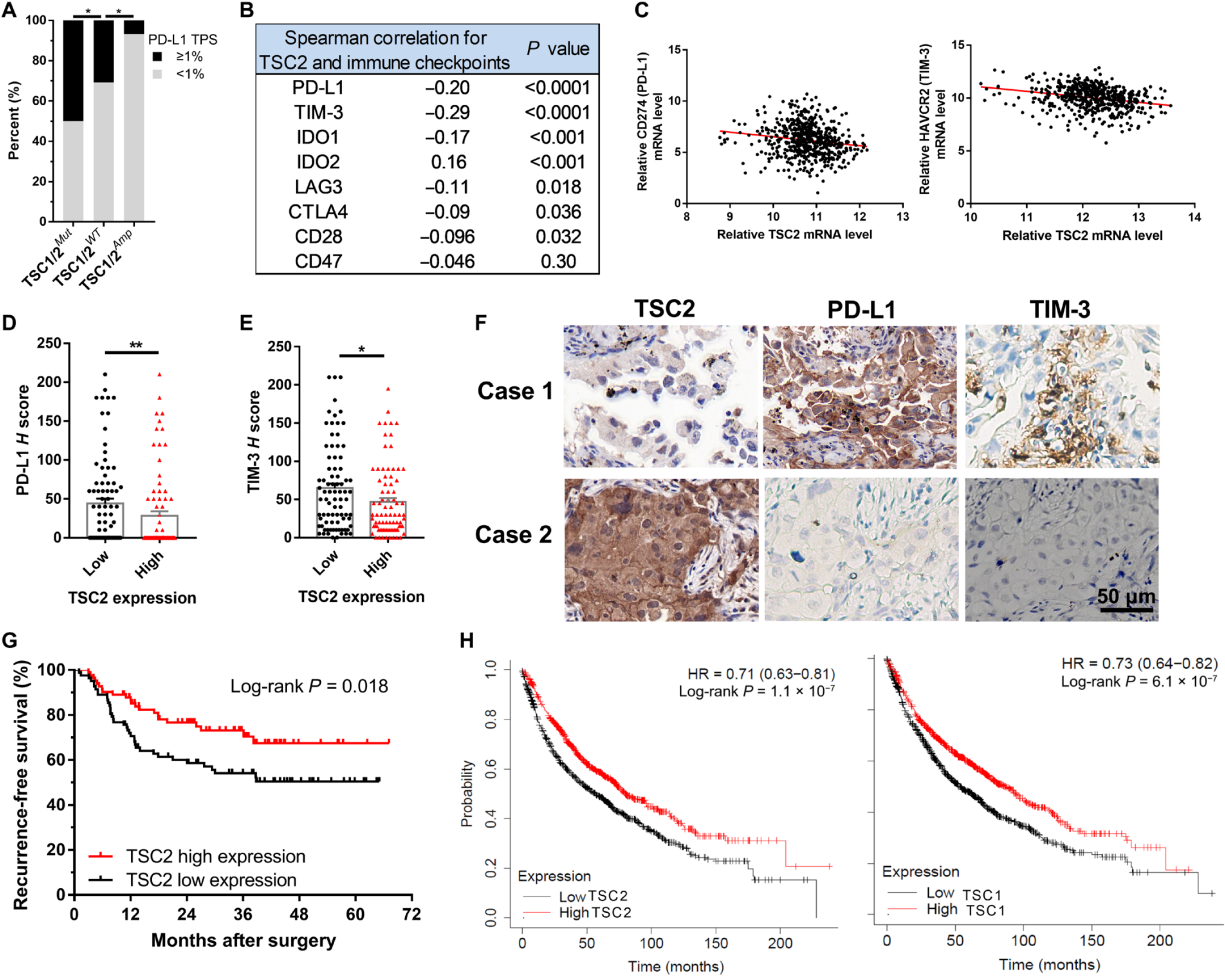
Correlation of *TSC2* with immune checkpoints in patient samples. (**A**) Comparison of the PD-L1–positive (TPS ≥ 1%) rates of patients harboring *TSC1/TSC2*-mutant, *TSC1/TSC2*-WT, or *TSC1/TSC2*-amplificated tumors in the FUSCC NGS cohort. Fisher’s exact test. (**B**) Summary of Spearman correlations between TSC2 and immune checkpoints mRNA levels in TCGA dataset. (**C**) Spearman correlations between TSC2 and PD-L1 and TIM-3 mRNA levels in (B). (**D** and **E**) Correlations between TSC2 and PD-L1 (D) and TIM-3 (E) expression level assessed by *H* score of immunostaining in FUSCC TMA cohort. Data are shown as means ± SEM. (**F**) Representative images of immunohistochemistry (IHC) for TSC2, PD-L1, and TIM-3 in (D) and (E). (**G**) RFS stratified by TSC2 protein expression level in the FUSCC TMA cohort. (**H**) Association of TSC2 (215735_s_at) and TSC1 (209390_at) mRNA levels with overall survival (OS) in NSCLC datasets from the KM plotter. *P < 0.05 and **P < 0.01. IDO, Indoleamine 2,3-dioxigenase; LAG3, lymphocyte-Activation Gene 3; CTLA4, cytotoxic T-lymphocyte associated protein 4; HAVCR2, hepatitis A virus cellular receptor 2; HR, hazard ratio.

To investigate the association between *TSC1*/*TSC2* mutation status and PD-L1 expression level, we analyzed the data of the Fudan University Shanghai Cancer Center (FUSCC) NGS cohort of patients with NSCLC undergoing surgical resection. *TSC1*/*TSC2-*mutant NSCLC had significantly higher PD-L1–positive [tumor proportion score (TPS) ≥ 1%] rate (19 of 38, 50.0%) compared with that of *TSC1*/*TSC2*-WT tumors (255 of 827, 30.8%), while the rate of PD-L1–positive in *TSC1*/*TSC2*-amplificated tumors was significantly lower (1 of 15, 6.7%), as shown in Fig. 3A. In addition to PD-L1, the association between *TSC2* and other non–PD-L1 immune checkpoints in TCGA dataset was also analyzed and summarized in Fig. 3B. The results displayed significantly inverse correlation between the mRNA levels of *TSC2* and immune checkpoints, including PD-L1 and T cell immunoglobulin and mucin domain-containing protein 3 (TIM-3) (Spearman correlation, *P* < 0.0001; Fig. 3, B and C). Next, we constructed a tissue microarray (TMA) consisting of 164 patients with NSCLC undergoing complete surgical resection in FUSCC, whose clinical and pathological characteristics were displayed in table S1. Immunostaining of PD-L1 and *TSC2* revealed a remarkably higher *H* score of PD-L1 (*P* = 0.0098; Fig. 3D) and TIM-3 (*P* = 0.028; Fig. 3E) protein in the *TSC2*–low expression patients than that in *TSC2*–high expression patients, verifying the previous finding that *TSC2* loss was associated with up-regulation of PD-L1 and TIM-3. The representative images of immunostaining of *TSC2*, PD-L1, and TIM-3 in patients with NSCLC were displayed in Fig. 3F.

Last, we performed Kaplan-Meier (KM) analysis and found that the recurrence-free survival (RFS) of patients with low expression of *TSC2* was significantly inferior to that of high expression of *TSC2* (3-year RFS, 54.1% versus 73.1%; log-rank test, *P* = 0.018; Fig. 3G). Moreover, the KM plotter (http://kmplot.com/analysis/) revealed that low mRNA levels of *TSC2* or *TSC1* were significantly associated with decreased overall survival of patients with NSCLC, respectively (Fig. 3H). The immunosuppressive TME of tumors with *TSC2* loss impaired the antitumor immune response, which could be an important reason for the unfavorable prognosis of these patients.

### *TSC2* loss is associated with an inflamed microenvironment in lung cancer patient samples

**Fig. 4.**
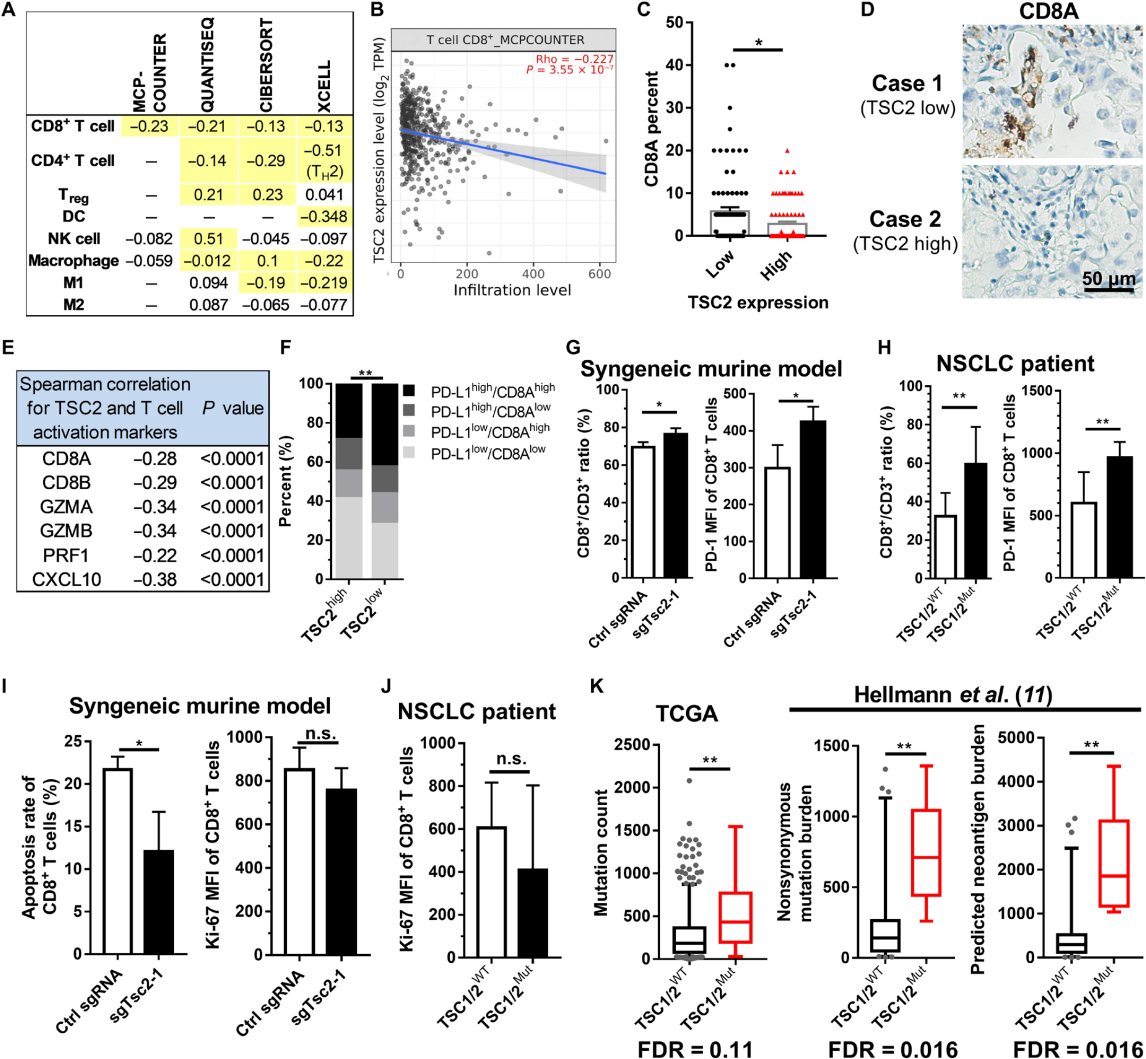
TSC2 loss reprogrammed TME. (**A**) Correlation between TSC2 level and immune filtrates. Data were derived from TIMER 2.0 (http://timer.comp-genomics.org/). Yellow highlight indicates statistical significance. T_reg_, regulatory T cell; NK, natural killer; T_H_2, T helper cell 2; DC, dendritic cell. (**B**) Correlation between TSC2 mRNA level and CD8^+^ T cell by MCP-COUNTER in (A). (**C**) Correlations between TSC2 and CD8A expression level assessed by *H* score of immunostaining in FUSCC TMA cohort. Data are shown as means ± SEM. (**D**) Representative images of CD8A in (C). (**E**) Summary of correlation between TSC2 and T cell activation markers level in TCGA dataset. (**F**) Comparison of immune phenotypes classified on the basis of PD-L1 and CD8A level by TSC2 level in TCGA dataset. (**G**) Bar graphs comparing the CD8^+^/CD3^+^ ratio and PD-1 MFI in CD8^+^ T cells in KP syngeneic murine models (*n* = 4 for each group). (**H**) Bar graphs comparing the CD8^+^/CD3^+^ ratio and PD-1 MFI in CD8^+^ T cells in patients with NSCLC (*TSC1/TSC2*-mutant, *n* = 5; *TSC1/TSC2*-WT, *n* = 15). (**I**) Bar graphs comparing the apoptosis rate and Ki-67 MFI in CD8^+^ T cells in KP syngeneic murine models. (**J**) Bar graphs comparing Ki-67 MFI in CD8^+^ T cells in patients with NSCLC. (**K**) Higher mutational load and predicted neoantigen burden of *TSC1/TSC2*-mutant patients in TCGA and Hellmann’s datasets. Box and whisker plots indicate median and 95% confidence interval. *P < 0.05 and **P < 0.01. TPM, transcripts per million.

To systematically depict the TME of *TSC2*-loss NSCLC, we used the TIMER 2.0 to evaluate the association between *TSC2* mRNA level and major immune cell subtypes by various algorithms. As summarized in Fig. 4A, *TSC2* mRNA level was negatively correlated with the infiltration of CD8^+^ T cell, CD4^+^ T cell, regulatory T cell, dendritic cell, natural killer cell, and macrophage (tumor purity adjustment). Notably, the correlation with CD8^+^ T cell was statistically significant by all the four algorithms we used. The scatter plot derived from MCP-counter was displayed in Fig. 4B. To confirm the correlation between *TSC2* and CD8^+^ T cell as the TIMER 2.0 revealed, we performed immunohistochemistry (IHC) in the TMA of 164 patients with NSCLC and found that *TSC2*–low expression tumors also exhibited remarkable increases in CD8A (*P* < 0.05; Fig. 4, C and D). In addition, *TSC2* showed a significantly negative correlation with the level of T cell activation markers, including CD8A, CD8B, granzyme A (GZMA), granzyme B (GZMB), perforin 1 (PRF1), and C-X-C motif chemokine ligand 10 (CXCL10) in TCGA database (Fig. 3E). It has been proposed to classify the TME as four different types based on the presence or absence of PD-L1 expression and tumor-infiltrating lymphocyte (TIL) (*9*, *10*). To further investigate the effects of *TSC2* status on TME, we analyzed the TCGA dataset and defined high *TSC2*, PD-L1, and CD8A as above-median expression. The proportion of PD-L1^high^/CD8A^high^ was 41.4% in the *TSC2*^low^ group, significantly higher than the 28.2% in the *TSC2*^high^ group (*P* < 0.01; Fig. 4F), indicating that *TSC2*-loss tumor displayed an inflamed TME, which had been demonstrated to be associated with benefits from immunotherapy (*9*, *10*).

We further performed flow cytometry in tumors from KP syngeneic murine models and patients with lung cancer. Compared with control group, KP-*Tsc2*-KO tumors had significantly increased percentage of CD8^+^ T cells (*P* < 0.01) and higher PD-1 mean fluorescence intensity (MFI) of CD8^+^ T cells in KP syngeneic murine models (*n* = 4 for each group, *P* < 0.05; Fig. 4G). In a prospectively performed cytometry of tumors from patients with NSCLC, we identified five tumors harboring mutations in *TSC1* or *TSC2*, and these tumors also had significantly increased CD8^+^/CD3^+^ ratio and higher PD-1 MFI of CD8^+^ T cells than *TSC1*/*TSC2-*WT tumors (*n* = 15, *P* < 0.01; Fig. 4H).

Then, we investigated the mechanisms of increased tumor-infiltrating CD8^+^ T cells in *TSC1*/*TSC2*-mutant tumors. In the KP syngeneic murine models, the apoptosis rate of CD8^+^ T cells was significantly reduced in the KP-*Tsc2*-KO tumors (*P* < 0.05), while the Ki-67 MFI was similar between two groups (Fig. 4I). Since apoptosis assay was not included in the protocol of cytometry for NSCLC patient samples, we failed to compare the apoptosis. The proliferation level of CD8^+^ T cells was comparable between *TSC1*/*TSC2*-mutant and *TSC1*/*TSC2-*WT NSCLC (Fig. 4J). The findings indicated that the apoptosis of CD8^+^ T cells was inhibited in the *TSC1*/*TSC2*-loss tumors.

Last, we analyzed the mutation count of TCGA dataset and revealed remarkably higher mutational load of *TSC1*/*TSC2*-mutant tumors [*P* < 0.01, false discovery rate (FDR) = 0.11; Fig. 4K]. We further reanalyzed the data of 75 patients with NSCLC from Hellmann’s dataset (*11*) and found an increase in both nonsynonymous mutation burden and predicted neoantigens in *TSC1*/*TSC2*-mutant tumors (both *P* < 0.01, FDR = 0.016; Fig. 4K). Collectively, *TSC2* loss is associated with the tumor mutational burden (TMB) and facilitated T cell activation in NSCLC.

### *TSC1*/*TSC2*-loss tumors benefit from immunotherapy in murine syngeneic model and patient cohort

**Fig. 5.**
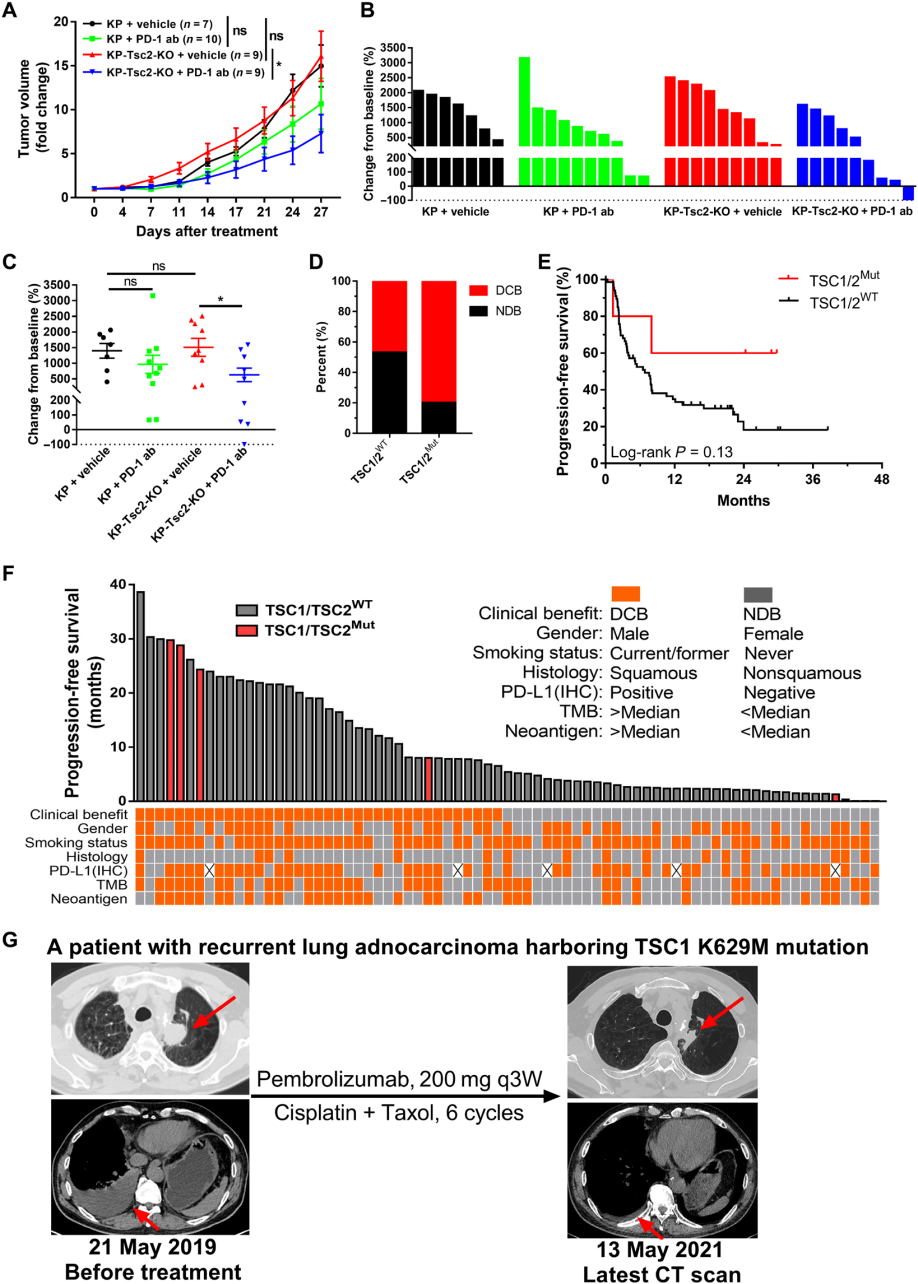
TSC1/TSC2-loss lung cancer benefits from ICB in syngeneic murine model and patients. (**A**) Anti–PD-1 antibody treatment in KP ctrl or KP-Tsc2-KO allograft tumors in subcutaneous injection model. Data are shown as means ± SEM and compared by repeated-measures analysis of variance (ANOVA). (**B**) Water fall plot showing tumor volume changes in response to treatment in (A). Each bar represents one tumor. (**C**) Tumor volumes at the treatment endpoint in (A). Data are shown as means ± SEM. (**D** and **E**) TSC1/TSC2 mutation was associated with higher durable clinical benefit (DCB) rate (D) and progression-free survival (PFS) (E) in 75 patients with NSCLC receiving immunotherapy. NDB, no durable benefit. (**F**) Individual PFS of 75 patients with NSCLC coupled with their clinical benefit, gender, smoking status, histology, TMB, and neoantigen burden in each patient. (**G**) Computed tomography (CT) images of a patient with recurrent *TSC1*-mutant lung adenocarcinoma before and after treatment with pembrolizumab and chemotherapy. q3W, every 3 weeks. *P < 0.05.

Previous findings showed that *TSC1*/*TSC2* loss identifies a subgroup of patients with NSCLC with poor prognosis and remains as an undruggable target (*12*). Tumors with inflamed TME, which was characterized with the presence of TIL and PD-L1 expression, had been demonstrated to be more likely to respond to ICB therapy. Hence, we used a KP murine syngeneic lung cancer model to test the hypothesis that *Tsc2*-deficient tumor was more sensitive to immunotherapy. For *Tsc2*-intact tumors, there was a modest inhibition of tumor growth by anti–PD-1 antibody treatment, without statistical significance. For *Tsc2*-KO tumors, anti–PD-1 antibody administration remarkably decreased tumor growth compared with vehicle (*P* < 0.05; Fig. 5, A to C). It is noteworthy that one *Tsc2*-KO tumor completely regressed after anti–PD-1 antibody treatment (Fig. 5, B and C).

Next, we analyzed the data of 75 patients with NSCLC from the CheckMate-012 trial by Hellmann *et al.* (*11*). Among five patients harboring mutations of *TSC1* and/or *TSC2*, four patients showed durable clinical benefit, while only 47% of patients with *TSC1*/*TSC2*-WT tumors show clinical benefit (Fig. 5D). Moreover, mutations of *TSC1* and/or *TSC2* were associated with superior progression-free survival, although statistical significance was not reached because of the limited sample size (Fig. 5E). Individual progression-free survival (PFS) of 75 patients with NSCLC coupled with their clinical benefit, gender, smoking status, histology, TMB, and neoantigen burden was summarized in Fig. 5F.

Last, we retrieved the patients receiving anti–PD-1/PD-L1 immunotherapy and identified one patient harboring *TSC1* K629M mutation. This patient, male, 68 years old, and former smoker, underwent video-assisted thoracoscopic surgery left upper lobectomy and mediastinal lymphadenectomy in November 2013, and histology examination revealed solid predominant adenocarcinoma (pT1bN0M0). In March 2019, he was diagnosed with disease relapse in left lung and right pleura, which was confirmed by cytopathology. He began treatment with 200 mg of pembrolizumab every 3 weeks combined with six cycles of cisplatin and taxol in May 2019 and showed a durable partial response for 29 months, with no evidence of progression or active disease at the latest follow-up in October 2021 (Fig. 5G). These findings suggested that *TSC1*/*TSC2*-loss patients with lung cancer might benefit from ICB therapy, despite the aggressive tumor biological behavior and unfavorable prognosis.

## DISCUSSION

Through in vitro and in vivo CRISPR screening in a KP lung cancer model, we identified *Tsc1* and *Tsc2* as regulators of antitumor immunity and sensitivity to ICB therapy. *TSC1*/*TSC2*-deficient tumors displayed a distinct inflamed TME, exhibiting as remarkably increase in expression of immune checkpoints and accumulation of T cells. *TSC1*/*TSC2* loss represented as a state of adaptive immune resistance and a high immunogenicity and, consequently, served as a potential biomarker of benefiting from ICB therapy.

The *TSC1*/*TSC2* complex integrates signals of multiple signaling pathways and controls cell metabolism and growth via regulating the mTOR pathway (*13*). *TSC1* and *TSC2* function as TSGs, and their mutations are common genetic events in NSCLC, with a frequency of 2 to 7%. Most of these mutations are inactivating mutations and lead to loss of protein expression (*14*), which makes *TSC1*/*TSC2* a poor therapeutic target in NSCLC. Our CRISPR screening study revealed the links between *TSC1*/*TSC2* mutations and antitumor immunity. Hence, in the present study, we performed comprehensive analyses and revealed the inflamed TME of *TSC1*/*TSC2*-loss NSCLC.

In vitro screening showed that the sgRNAs of *Tsc1* and *Tsc2* were significantly enriched in the Pd-l1–high subpopulation, indicating that *Tsc1*/*Tsc2* loss promoted Pd-l1 expression. This finding was validated in both lung cancer cell lines and patient samples. In particular, we analyzed an NGS cohort and a TMA IHC cohort of patients with NSCLC undergoing surgical resection at FUSCC and demonstrated that both mutation status and protein expression of *TSC1*/*TSC2* were significantly associated with PD-L1 expression. Furthermore, we found that loss of *TSC2* up-regulated the PD-L1 of lung cancer cells at the transcriptional level. By contrast, Lastwika *et al.* (*15*) showed that mTOR, the downstream target of *TSC1/TSC2*, increased PD-L1 expression at the translational level. This indicates that *TSC1/TSC2* may control PD-L1 expression through both mTOR-dependent and mTOR-independent pathways, at both mRNA and protein levels.

In addition to up-regulating PD-L1 expression, inactivation of *TSC1*/*TSC2* also reprogrammed the immune microenvironment by increasing the recruitment of CD8^+^ T cells. PD-L1 expression induced by *TSC1*/*TSC2* deficiency inhibited the cytotoxic effects of T cells. We also found that *TSC1*/*TSC2* mutations enhanced the expression levels of some other immune-inhibitory checkpoints, including PD-1 and TIM-3. The infiltrating CD8^+^ T cells were in an exhausted state mediated by these checkpoints, resulting in an immunosuppressive microenvironment and accomplishing immune evasion of the tumor cells. As TIM-3 up-regulation has been reported to mediate resistance to anti–PD-1 immunotherapy (*16*), combinational therapies blocking both PD-1 and TIM-3 pathways may be particularly beneficial to *TSC1*/*TSC2*-deficient tumors. Further investigations are warranted in future preclinical studies and clinical trials.

Our analysis also found that *TSC1*/*TSC2*-mutant NSCLC had significantly higher TMB. Multiple studies have shown that TMB may be a surrogate for overall neoantigen load and is robustly predictive of clinical benefit to ICB (*11*, *17*). Previous studies have shown the interplay between *TSC1*/*TSC2* loss and DNA damage response (DDR) system. Mechanistically, loss of *TSC2* hyperactivates mTORC1 signaling and decreases the abundance of RNF168 protein, which is involved in DNA double-strand break repair, resulting in defects in the DDR and accumulation of genetic mutations (*18*).

Collectively, our data derived from cell lines, syngeneic mouse models, and specimen of patients with NSCLC revealed the prominent significance of *TSC1*/*TSC2* mutations in promoting PD-L1 expression, facilitating T cell infiltration and augmenting tumor immunogenicity. This work provided evidence that *TSC1*/*TSC2*-mutant NSCLC could benefit from ICB therapy, highlighting the predictive value of *TSC1*/*TSC2* status in guiding immunotherapy.

## MATERIALS AND METHODS

### Cell culture and lentivirus infection

Human embryonic kidney (HEK) 293T cell line was cultured in Dulbecco’s modified Eagle’s medium (Gibco) with 10% fetal bovine serum (FBS). Murine Kras^G12D^/p53^−/−^ (KP) lung cancer cell line (C57BL/6 background) was established and characterized as described previously (*19*) and cultured in RPMI 1640 (Gibco) with 10% FBS.

Lentiviral package and infection were done as follows: HEK-293T cells were cotransfected with pLenti-Cas9-puro, pXPR-GFP-Blast-sgRNA, or lentiCRISPR v2 construct and packaging plasmids PSPAX2 and PMD2.G using Lipofectamine 3000 (Invitrogen). The progeny viruses released from HEK-293T cells were filtered by 0.45-μm filter (Corning), and polybrene (Sigma-Aldrich) was added to the cell culture medium containing viral particles to increase the infection efficacy. Stable cell lines were selected and maintained in puromycin (2 μg/ml) or blasticidin (5 μg/ml). For rescue experiment, the plasmid pcDNA3.1-h*TSC2* was purchased from Shanghai Asia–Vector Biotechnology Co. Ltd. (catalog no. 2497). Mouse *Tsc2* gene has a high degree of homology with human (*20*), and the coding sequence of human TSC2 does not contain the target sequences of the three sgRNA for mouse *Tsc2*.

### CRISPR screening

The construction of a pooled lentiviral sgRNA library, which included *Tsc1* and *Tsc2*, for CRISPR screening was previously described (*6*). There were 8 to 12 sgRNAs for each gene in the library. Two KP-Cas9 clones (clones 7 and 9), whose Cas9 expression was confirmed by Western blot (WB), were transduced at a multiplicity of infection of 0.2 with lentivirus produced from the library with at least 1000-fold coverage. For in vitro screening, we used flow cytometry to sort the top 15% PD-L1–high populations and top 15% PD-L1–low populations of transduced KP cells after 2-week culture in dishes. For in vivo screening, the transduced KP cells were subcutaneously transplanted to C57BL/6 mice and treated with anti–PD-1 antibody or isotype control (three times per week) on day 7; tumors were harvested on day 24 (*6*). The sorted cell pools and harvested tumors were subjected for genomic DNA extraction (DNA Blood Midi Kit, QIAGEN), sgRNA amplification, and NGS on an Illumina HiSeq to determine sgRNA abundance. The target sequences of sgRNA are displayed in table S2.

### Flow cytometry

One million cells per condition were harvested and resuspended in phosphate-buffered saline (PBS) with 2% FBS. The cell pellets were stained with the PD-L1 antibody (clone 10F.9G2, BioLegend) diluted in PBS and 2% FBS for 30 min on ice. Cells were imaged on a BD Biosciences LSRFortessa and analyzed with FlowJo software.

### Real-time quantitative polymerase chain reaction

qRT-PCR was conducted as previously described (*6*) using StepOnePlus Real-Time PCR System (Applied Biosystems). All reactions were performed in triplicate. Primer sequences were as follows: Pd-l1 (mouse), 5′-ACTTGCTACGGGCGTTTACT (forward) and 5′-ACTAACGCAAGCAGGTCCAG (reverse); actin (mouse), 5′-CTGTCCCTGTATGCCTCTG (forward) and 5′-ATGTCACGCACGATTTCC (reverse).

### WB and IHC

Cells were lysed with radioimmunoprecipitation assay buffer containing protease/phosphatase inhibitor cocktail (Thermo Fisher Scientific) on ice. Protein lysates were separated by 4 to 12% bis-tris gels (Invitrogen) and then transferred to a polyvinylidene difluoride membrane. After the membranes were blocked, they were immunoblotted at 4°C overnight with antibodies against TSC2 [D93F12, Cell Signaling Technology (CST)] and β-actin (Ab8227, Abcam). IRDye 800–labeled goat anti-rabbit immunoglobulin G (IgG) and IRDye 680–labeled goat anti-mouse IgG secondary antibodies were purchased from LI-COR Biosciences, and membranes were detected with an Odyssey detection system (LI-COR Biosciences).

We constructed a cohort consisting of 880 patients with NSCLC whose resected specimen was profiled by targeted NGS at FUSCC (FUSCC NGS cohort). We retrieved the clinicopathologic information, including PD-L1 TPS (22C3) from the medical record, and compared PD-L1 TPS across the mutation status of *TSC1*/*TSC2*. Besides, a TMA of 164 patients with NSCLC who underwent complete surgical resection was constructed with 1.5-mm core from one tumor block of each of the resection specimen (FUSCC TMA cohort). Tissue sections prepared for antigen retrieval by microwave treatment in citrate buffer (pH 6.0) were incubated with anti-TSC2 (D93F12, CST), anti–PD-L1 (E1L3N, CST), anti–TIM-3 (D5D5R, CST), and anti-CD8A (D8A8Y, CST) primary antibodies. Immunostaining was performed with diaminobenzidine solution under a microscope and counterstained with hematoxylin.

This study was approved by the Institutional Review Board of the FUSCC. Informed consent was obtained from each patient.

### In vitro killing assay

We genetically generated a KP-Ova cell line. The KP-Ova-*Tsc2*-KO cells or KP-Ova-Ctrl cells were cocultured with OT-1 T cells in a 1:3 ratio (0.05 million tumor cells:0.15 million OT-1 T cells) in 96-well plates. After coculture for 2 days, we washed out OT-1 T cells with PBS and then used Cell Counting Kit-8 (ALX-850-039-KI02, Enzo) to measure tumor cell activity. The control was performed as 0.05 million KP-Ova-*Tsc2*-KO cells or KP-Ova-Ctrl cells cultured in the absence of OT-1 T cells for 2 days. The relative cell activity = optical density (OD) average of tumor cells in the presence of OT-1 T cell/OD average of tumor cells in the absence of OT-1 T cell × 100%.

### Mouse treatment study

All mouse work was reviewed and approved by the Institutional Animal Care and Use Committee at NYU School of Medicine or Dana-Farber Cancer Institute. Specific pathogen–free facilities were used for housing and care of all mice. Cells were injected subcutaneously into 6- to 8-week-old C57BL/6 mice (the Jackson Laboratory). Mouse anti–PD-1 antibody (29F.1A12) (*21*) or isotype control was administered three times per week (Monday, Wednesday, and Friday) at 200 μg per mouse via intraperitoneal injection. Tumor maximal length and width were measured by calipers twice a week. Tumor volume was calculated with the following formula: (*L* × *W*^2^)/2.

### Statistical analysis

All statistical analyses were performed using GraphPad Prism 7. Data were analyzed by Student’s *t* test or Mann-Whitney *U* test as appropriate. Survival curve was plotted with KM method and compared by log-rank test. Level 3 data for TCGA were obtained through the TCGA portal and were sorted on the basis of the mutation status or expression level of *TSC1* and *TSC2*. Correlation of mRNA expression levels and patient survival in NSCLC was done using the KM plotter (http://kmplot.com/analysis/) (*22*). *P* < 0.05 (two-tailed) was considered as statistically significant (*P < 0.05, **P < 0.01, ***P < 0.001, and ****P < 0.0001).

## Supplementary Material

20220202-1

## Notes

**Correction (23 March 2022):** In Figure 1A the authors mistakenly wrote “B57BL/6” in reference to the mouse strain used, instead of “C57BL/6.” This has now been corrected.

## Notes

View/request a protocol for this paper from *Bio-protocol*.
