# Supplementary material for: Loss of TSC1/TSC2 sensitizes immune checkpoint blockade in non–small cell lung cancer
Source: Sci Adv. 2022 Feb 4;8(5):eabi9533. doi: 10.1126/sciadv.abi9533 (PMC8816329; doi:10.1126/sciadv.abi9533)

Science Advances
AAAS

Supplementary Materials for

Loss of *TSC1/TSC2* sensitizes immune checkpoint blockade in non–small cell lung cancer

Qingyuan Huang, Fei Li*, Hai Hu, Zhaoyuan Fang, Zhendong Gao, Guozhan Xia, Wai-Lung Ng, Alireza Khodadadi-Jamayran, Ting Chen, Jiehui Deng, Hua Zhang, Christina Almonte, Kristen Labbe, Han Han, Ke Geng, Sittinon Tang, Gordon J. Freeman, Yuan Li, Haiquan Chen*, Kwok-Kin Wong*

*Corresponding author. Email: hqchen1@yahoo.com (H.C.); kwok-kin.wong@nyulangone.org (K.-K.W.); li_fei@fudan.edu.cn (F.L.)

Published 04 February 2022, *Sci. Adv.* **8**, eabi9533 (2022)
DOI: 10.1126/sciadv.abi9533

This PDF file includes:

Tables S1 and S2
Figs. S1 to S6

Supplementary Table S1. Baseline clinical and pathological characteristics of 164 NSCLC patients in the FUSCC TMA cohort

Parameters	TSC2 expression level		*P* value
	Low (%)	High (%)	
**Age**			0.87
≤60 yr	35(42.7)	33 (40.2)	
>60 yr	47(57.3)	49 (59.8)	
**Gender**			1.00
Female	41(50)	40(48.8)	
Male	41(50)	42(51.2)	
**Smoking history**			0.63
Never	54(65.9)	50(61)	
Current/Former	28(34.1)	32(39)	
**Pathologic stage**			0.71
I	50(61)	55(67.1)	
II	10(12.2)	10(12.2)	
III	22(26.8)	27(20.7)	
**Histologic subtype**			0.57
AIS/MIA/lepidic	9(11)	11(13.4)	
Acinar/papillary	49(59.8)	52(63.4)	
solid/micropapillary/IMA	24(29.2)	19(23.2)	

Supplementary Table S2. Target sequences of sgRNA

Gene	Target sequence (5'-3')
Mouse TSC1-sgRNA1	CTCCCGATGATCCCGCAGTC
Mouse TSC1-sgRNA2	TTCCCTGACTGCGGGATCAT
Mouse TSC2-sgRNA1	TGCTAGCAGCATCCGACTAC
Mouse TSC2-sgRNA2	ATAAGGTGAGAACTGCATGC
Mouse TSC2-sgRNA3	TGACGAATACATTGCATCAA
Human TSC2-sgRNA1	GGTCGCGGATCTGTTGCAGC
Human TSC2-sgRNA3	GCTGAAGGCCATCGTGCAG

Supplementary Figure S1. Sanger sequencing of genomic DNA shows efficient genome editing of KP cell pools transfected with lentiviral gRNA targeting *Tsc2*.

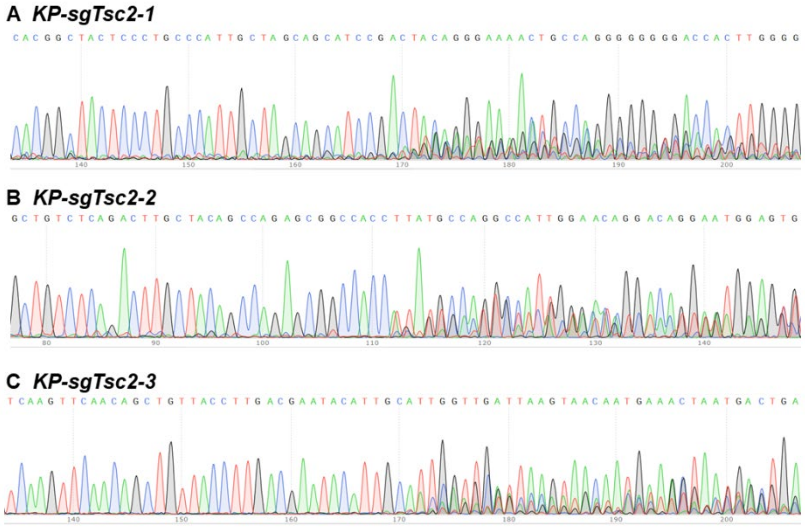

Supplementary Figure S2. Sanger sequencing of genomic DNA shows efficient genome editing of KP clones transfected with lentiviral gRNA targeting *Tsc2*.

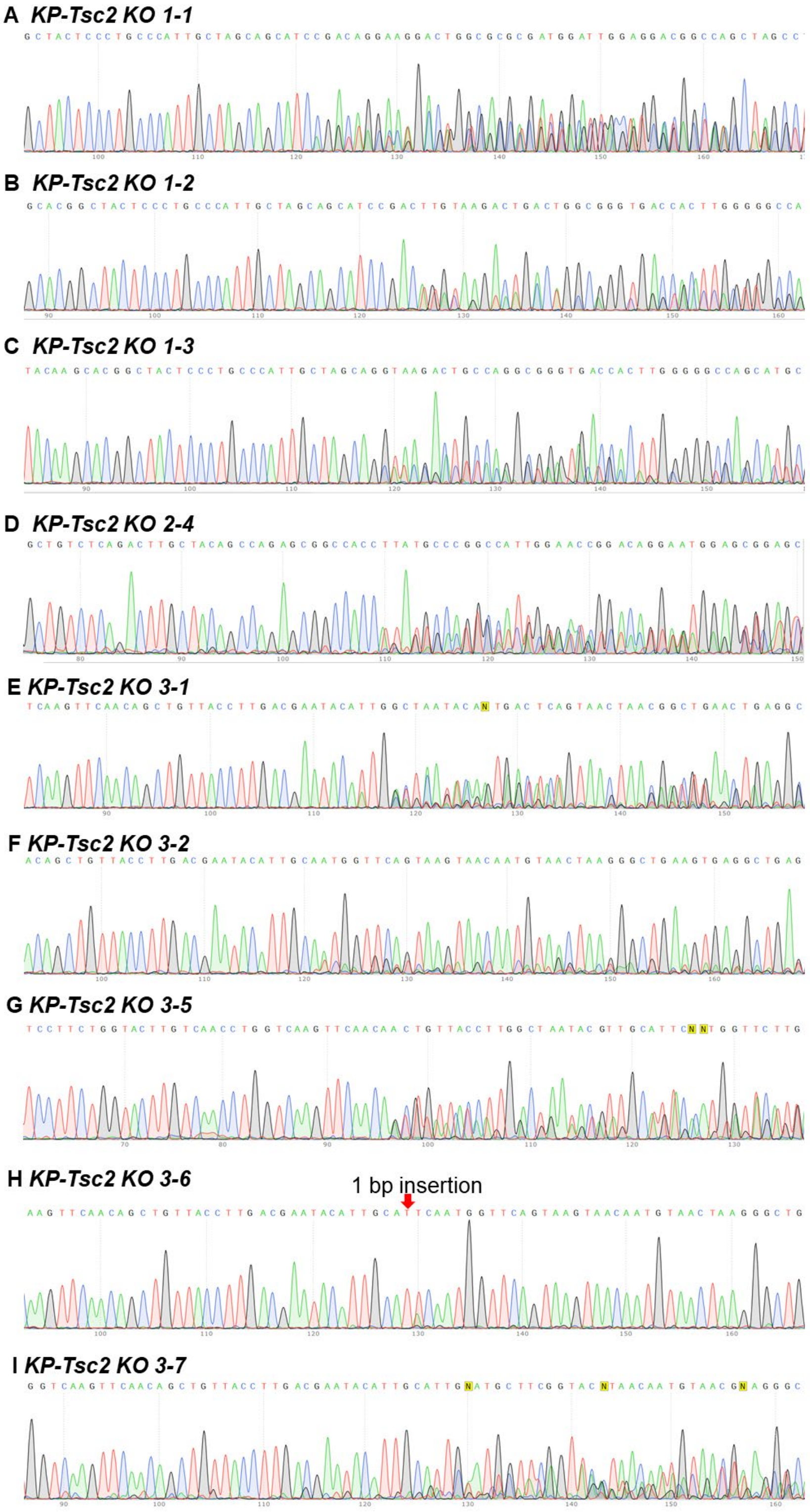

Supplementary Figure S3. Sanger sequencing of genomic DNA shows efficient genome editing of A549 clones transfected with lentiviral gRNA targeting TSC2.

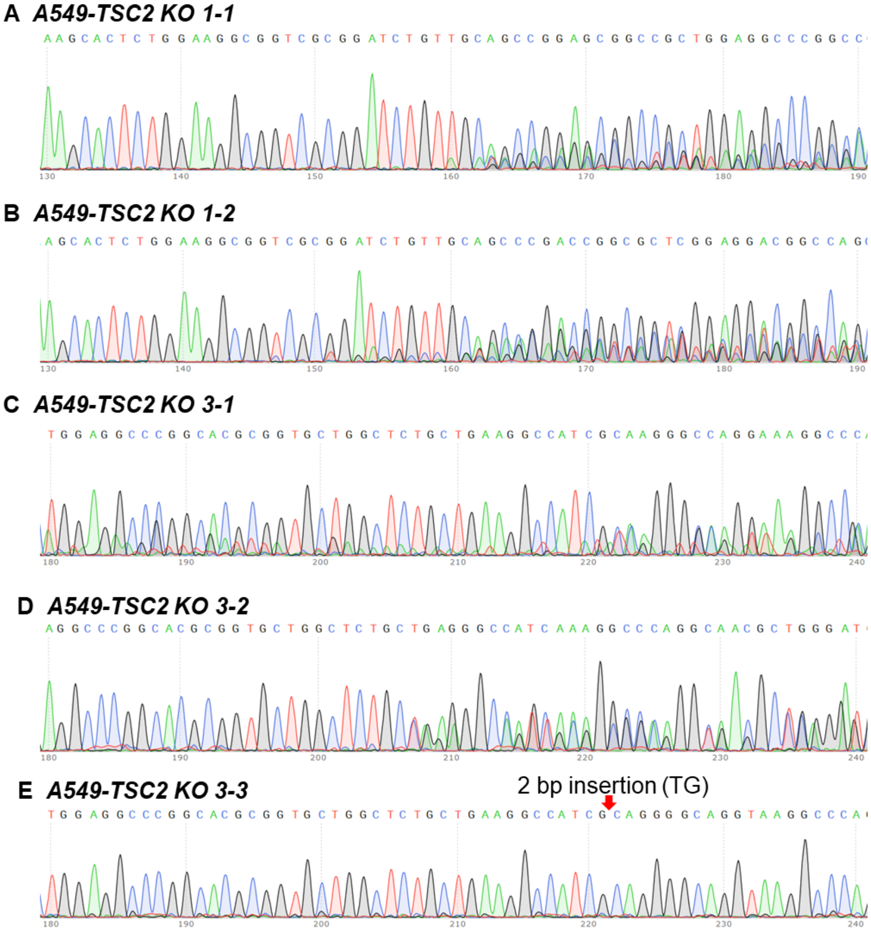

Supplementary Figure S4. Western blot analyses of KP-Tsc2-KO cell lines transfected with vehicle or human TSC2.

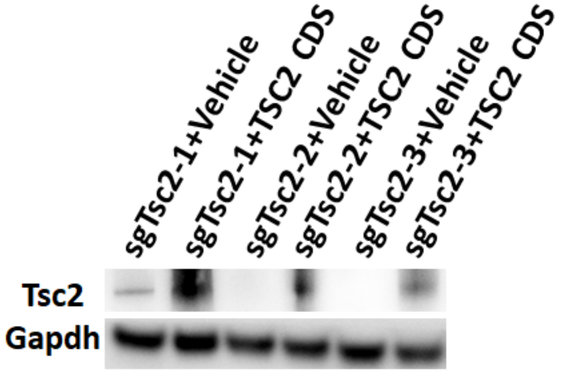

Supplementary Figure S5. Sanger sequencing of genomic DNA shows efficient genome editing of KP cell pools transfected with lentiviral gRNA targeting *Tsc1*.

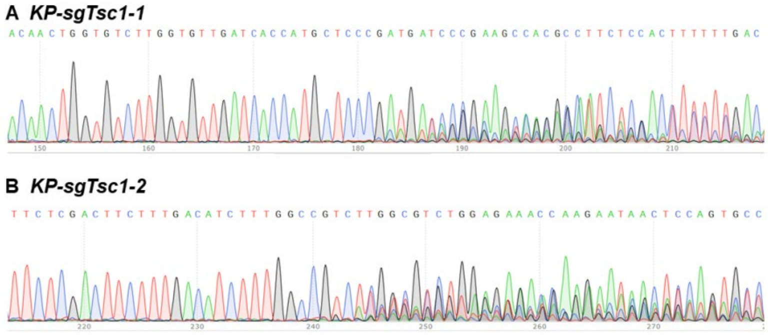

Supplementary Figure S6. Western blot analyses of Tsc2 expression in the control or *Tsc2*-KO KP-Ova cells in the *in vitro* killing assay.

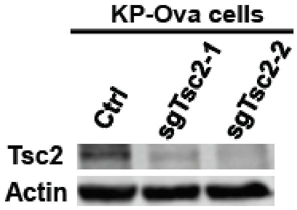

Supplement: Supplementary file 2 — Tables S1 and S2 Figs. S1 to S6 [file sciadv.abi9533_sm.pdf]
